# Neighbourhood safety and leisure-time physical activity among Dutch adults: a multilevel perspective

**DOI:** 10.1186/1479-5868-10-11

**Published:** 2013-01-28

**Authors:** Daniëlle Kramer, Jolanda Maas, Marleen Wingen, Anton E Kunst

**Affiliations:** 1 Department of Public Health, Academic Medical Centre, University of Amsterdam, PO Box 22660, Amsterdam, 1100 DD, The Netherlands; 2 Department of Public and Occupational Health, and the EMGO Institute for Health and Care Research, VU University Medical Centre, Amsterdam, the Netherlands; 3 Department of Social and Spatial Statistics, Statistics Netherlands, Heerlen, the Netherlands

**Keywords:** Physical activity, Walking, Cycling, Safety, Crime, Environment

## Abstract

**Background:**

Several neighbourhood elements have been found to be related to leisure-time walking and cycling. However, the association with neighbourhood safety remains unclear. This study aimed to assess the association of neighbourhood-level safety with leisure-time walking and cycling among Dutch adults.

**Methods:**

Data were derived from the national health survey (POLS) 2006–2009, with valid data on 20046 respondents residing in 2127 neighbourhoods. Multilevel logistic regression models were used to examine the association between neighbourhood-level safety (general safety and specific safety components: physical disorder, social disorder, crime-related fear, traffic safety) and residents’ engagement in outdoor leisure-time walking and cycling for at least 30 minutes per week.

**Results:**

An increase in neighbourhood safety (both general safety and each of the safety components) was significantly associated with an increase in leisure-time cycling participation. Associations were strongest for general safety and among older women. In the general population, neighbourhood safety was not significantly associated with leisure-time walking. However, among younger and older adult men and lower educated individuals, an increase in general safety was associated with a decrease in leisure-time walking participation.

**Conclusions:**

In the Netherlands, neighbourhood safety appears to be related to leisure-time cycling but not to walking. Leisure-time cycling may best be encouraged by improving different safety components at once, rather than focusing on one safety aspect such as traffic safety. Special attention is needed for older women.

## Background

Leisure-time walking and cycling may be appropriate types of physical activity (PA) to achieve current PA recommendations, since they are easy to implement in daily life with low cost and little risk of injury. The most effective ways to encourage these types of PA in adults are yet uncertain. Traditionally, focus has been on individual-level determinants of PA. Recently, a complementary ecological approach has been taken on which postulates that PA is also influenced by the individuals’ living environment [1].

To create neighbourhood environments that encourage leisure-time walking and cycling, it is important to understand which neighbourhood elements are most strongly related to these types of PA. According to a recent review, no firm conclusions can be drawn on environmental determinants of cycling due to lack of studies on this topic [2]. Leisure-time walking has been studied more extensively. Pedestrian infrastructure and neighbourhood aesthetics have consistently been found to be associated with leisure-time walking [3]. There is less consistent empirical support for other neighbourhood elements.

One of these elements is neighbourhood safety. Some studies have found a positive association between general neighbourhood safety and walking in leisure time [4,5], but most studies did not find an association [6-13]. Many of the studies on neighbourhood safety have used a composite measure, in which various safety components (e.g. traffic, crime, disorder) are combined into one comprehensive safety variable [4,6,7,9-12]. The use of such combined safety indicators might obscure the effects of the specific safety components.

Studies on specific safety components have most often explored the role of traffic safety. Inoue et al. [14] found that people who perceived good traffic safety were 1.5 times more likely to walk in leisure-time than those who perceived poor traffic safety. However, much other research has found inconsistent [5,15,16] or no associations [6,7,17-20] between traffic safety and walking. With regard to crime safety, again, results are inconsistent. Shigematsu et al. [18] found respondents’ perceptions to be associated with leisure-time walking, but only in some age groups. Other studies have found no association at all [6,14,17,20,21]. Only few studies have explored the role of neighbourhood disorder. Cleland et al. [16] found limited evidence of a positive association with leisure-time walking. Absence of physical neighbourhood disorder was positively associated with maintaining high levels of leisure-time walking between baseline and follow-up, but not with three other leisure-time walking outcomes. Other studies did not find any significant association of leisure-time walking with indicators of neighbourhood disorder such as garbage, graffiti or public drunkenness [6,7].

Comparability of these results is limited, due to differences in settings, PA measures, and safety measures. Therefore, it is hard to determine the relative impact of each specific safety component on leisure-time walking behaviour. Studies are needed that simultaneously explore the association of various safety components with leisure-time walking. To our knowledge, there is only one American study that has explored multiple safety components [6]. The authors found no association between leisure-time walking and objective measures of criminal offenses, traffic-related offenses, physical disorder and social disorder.

As the latter study [6], most studies on safety and leisure-time walking have been performed in America and Australia [2,22]. European studies on this topic are rare. Yet, results may be different in Europe because PA patterns as well as the safety situation of neighbourhoods may differ [23,24]. For example, the association of leisure-time PA with crime and traffic safety may be less strong, because of safer traffic and lower crime rates in deprived neighbourhoods in Europe compared to America [23]. Within Europe, the Netherlands may be a particularly interesting country to explore the associations between neighbourhood safety and leisure-time PA. Due to high prevalence rates of walking and cycling [25], it offers the opportunity to explore the association of neighbourhood safety with both walking and the much less studied PA component cycling. The current evidence on the environmental correlates of cycling mainly comes from the transportation literature and has focused on cycling for transport rather than cycling in leisure time [17,20,26,27]. The one study on leisure-time cycling found no significant association with general safety [28].

The aim of the current study is to explore the association of neighbourhood-level safety with leisure-time walking and cycling in a large sample of Dutch adults. First, the association of general safety with leisure-time walking and cycling will be explored. Next, we will explore whether these associations are different for specific safety components. Following McGinn et al. [6], the safety components will include physical disorder, social disorder, crime-related fear and traffic safety. In a last step, we will explore whether associations differ by subpopulation. A review of Foster et al. [22] has postulated that associations may differ according to age, gender, education, and residential density of the neighbourhood. Women, elderly and lower educated tend to feel more vulnerable which may manifest itself in a stronger association of safety with leisure-time walking and cycling. Further, we postulate that in the Netherlands, densely populated areas may provide increased natural surveillance from both houses and pedestrians, which may make people feel less vulnerable to unsafe situations. If so, this would result in a weaker effect of safety on leisure-time walking and cycling in densely populated areas. Little research has focused on these differences.

## Methods

### Study population

Cross-sectional data on individual characteristics and leisure-time walking and cycling behaviour were obtained from the Dutch national health survey of 2006 to 2009, which is part of the yearly administered Dutch Integrated Survey on Household Living Conditions (POLS). A random nationwide sample of in total 57,281 non-institutionalized persons was drawn from the national population registry. Selected individuals were approached by an interviewer and asked to participate in an interview and, if 12 years and older, to fill in an additional paper-and-pencil survey on specific health topics, including PA. There was a non-response of 36% for the interview, with an additional 16% non-response for the survey. Due to the age restriction, 16% of the sample was not eligible to complete the paper-and-pencil survey. A total of 25.206 persons of 12 years and older completed the survey.

Cross-sectional data on residents’ perceptions of neighbourhood safety were obtained using the three-yearly conducted Dutch Housing Questionnaire (WoON) of 2006. A random nationwide sample of 113,837 non-institutionalized adults (18 years and older) was approached by phone and asked to complete an interview either by phone, face to face, or on paper. There was a non-response of 42%. A total of 64,005 adults from 3,495 neighbourhoods completed the survey. To assess the safety situation in each neighbourhood at large, we constructed neighbourhood-level safety scores by averaging the scores of all WoON res pondents living in the same neighbourhood. Neighbourhood-level safety data from the WoON survey were linked to individual-level data from the Dutch national health survey using the 4-digit zipcode.

From the POLS data, we excluded respondents younger than 18 (N= 2,237), respondents whose neighbourhood-level safety scores have been based on less than five observations in the WoON survey (N=2,369) and respondents with unrealistic and missing PA scores (N=554 for walking, N=566 for cycling). PA scores were classified as unrealistic if the score exceeded 3360 minutes per week, which equals 8 hours each day of the week. A total remained of 20,046 POLS respondents with valid walking scores and 20,034 respondents with valid cycling scores, living in 2,127 neighbourhoods.

### Measures

#### Leisure-time walking and cycling

Self-reported PA was measured in POLS using the Dutch Short QUestionnaire to ASsess Health-enhancing PA (SQUASH). This instrument has shown to be fairly reliable and valid, especially for large samples [29,30]. Respondents were asked to report the frequency (number of days) and duration (hours and minutes per day) of leisure-time walking and cycling and other PA activities in a typical week. Total minutes per week spent on walking and cycling in leisure time were calculated.

The distribution of leisure-time walking and cycling was highly skewed with 37% reporting zero minutes of leisure-time walking per week and 45% of respondents reporting zero minutes of cycling. Therefore, instead of using a continuous outcome measure, dummy variables were created for leisure-time walking and cycling by classifying respondents as ‘inactive’ (less than 30 minutes per week) versus ‘active’ (at least 30 minutes per week) levels. In other PA studies, respondents have often been classified on the basis of the WHO recommendation of 150 minutes per week. However, this 150 minutes cut-off point applies to overall levels of PA. In this study, we used a lower cut-off point because we focused on two specific components of PA (walking and cycling). Sensitivity analyses showed that the results of the present study are robust against alternative cut off points for leisure-time walking or cycling (0 or 60 minutes per week).

#### Neighbourhood safety

We measured four specific safety components, based on the distinctions made by McGinn et al. [6]: physical disorder, social disorder, crime-related fear and traffic safety.

•Physical disorder was assessed using the five items ‘graffiti on walls and buildings’, ‘devastation of phone booths and bus-/tram booths’, ‘rubbish on the street’, ‘dog faeces on the street’ and ‘smell, dust and/or dirt’. All items used a three-point Likert scale ranging from 1 (often) to 3 (never). The physical disorder scale was computed by taking the average score of these five items. Alpha reliability analyses have been performed to determine the internal consistency of this scale. Cronbach’s alpha was 0.73, indicating good reliability.

•Social disorder was assessed using the three items ‘nuisance from direct neighbours’, ‘nuisance from other neighbourhood residents’ and ‘nuisance from youth’. All items used a three-point Likert scale ranging from 1 (often) to 3 (never). A social disorder scale was computed by taking the average score of these three items. Cronbach’s alpha was 0.76, indicating good reliability.

•Crime-related fear was assessed using the one item ‘I am afraid to be troubled or robbed in this neighbourhood’. Answers were on a three-point Likert scale ranging from 1 (often) to 3 (never).

•Traffic safety was assessed using the one item ‘I think the traffic situation in this neighbourhood is safe’. This item used a five-point Likert scale ranging from 1 (totally agree) to 5 (totally disagree). The two upper and lower scores have been combined, resulting in a three point Likert scale ranging from 1 (agree) to 3 (disagree).

All four safety variables were coded such that a higher score indicated higher perceived safety. The general safety scale has been composed by computing the average score on the four safety components. Cronbach’s alpha was 0.68, indicating fair reliability.

The measures above were constructed for the individual respondents of the WoON survey. Neighbourhood-level safety variables were constructed for each of the five safety measures by computing the average of the safety scores of all respondents living within the same 4-digit zipcode. All five neighbourhood-level safety variables were measured as continuous variables ranging from 1 (not safe) to 5 (very safe).

#### Potential confounders

Potential individual-level confounders that have been measured are age (continuous), gender, household composition (five categories), ethnicity (four categories) and socioeconomic status (SES). Three indicators of SES were included: education (five ordinal groups), disposable household income (five quintiles of net income in Euros) and disposable household wealth (five quintiles of the assembly of assets and debts in Euros). Age, gender, household composition and education have been assessed using the POLS questionnaire. Ethnicity was derived from the national population registry. Information on household income and wealth was obtained from the national tax registration. A potential neighbourhood-level confounder that has been included is population density (five ordinal groups). Population density has been estimated for each neighbourhood using data on the address density of the wider municipality. Data on address density has been derived from Statistics Netherlands.

### Statistical methods

To take the sampling design of the POLS survey into account, prevalence of leisure-time walking and cycling were weighted for age, gender, marital status, household size, urbanization, province, and month of survey. The associations of (specific components of) neighbourhood safety with leisure-time walking and cycling were assessed by means of odds ratios derived from multilevel logistic regression analysis. Level 1 represented individuals, level 2 represented the neighbourhoods. A three-stage modelling approach was used to make a stepwise adjustment for age, gender, household composition, ethnicity (model 1), education, household income, household wealth (model 2) and population density (model 3). Each potential confounder was included as a set of dummy variables. Subpopulation analyses were performed using model 3. Potential effect-modification by age-gender, education and neighbourhood population density was investigated by entering interaction terms in the model. Intraclass Correlation Coefficient (ICC) was estimated using Rho to display the proportion of total variance in leisure-time walking and cycling that is attributable to the neighbourhood level. Associations were weighted using a complex survey sample design with sample weights. Results were almost identical to those presented below. For all analyses, statistical significance was set at 0.05. Analyses were carried out using the STATA 11.0 software.

This study was based on secondary analyses of anonymized data by Statistics Netherlands (the “CBS”), with approval of the CBS authority for privacy protection. This authority has the responsibility to guarantee that all activities of the CBS are in strict agreement the Dutch laws for the protection of privacy of residents and subjects.

## Results

Among the total adult population, the weighted prevalence of leisure-time walking was higher than of cycling (Table 1). More women than men engaged in leisure-time walking and cycling. Weighted prevalence of leisure-time walking and cycling among both groups increased with age up to 46–65 years old.

**Table 1:** Weighted prevalence of leisure-time walking and cycling among the valid study population and various subgroups

		**Leisure-time walking**		**Leisure-time cycling**	
**Population (years)**	**N with valid PA scores**	**% N ≥30 min/week**	**Mean min/week (SD)**	**% N ≥30 min/week**	**Mean min/week (SD)**
Total adult population	20046	61.9	158 (265)	52.5	131 (267)
Men 18-30	1492	42.8	97 (276)	42.8	97 (267)
Men 31-45	2542	58.5	121 (232)	48.8	90 (214)
Men 46-65	3557	64.6	169 (254)	54.1	140 (288)
Men > 65	1870	59.8	185 (266)	56.8	204 (331)
Women 18-30	1703	58.5	130 (268)	49.0	97 (227)
Women 31-45	2913	68.3	167 (272)	57.7	126 (266)
Women 46-65	3718	70.4	196 (289)	60.8	160 (276)
Women > 65	2251	58.7	172 (241)	40.8	128 (238)

These age and gender differences in PA were found to be significant in multilevel logistic regression analyses (Table 2). Moreover, non-natives, married people, and higher educated people were significantly more likely to walk and cycle in leisure-time compared to their counterparts. Leisure-time cycling was positively associated with household wealth, but negatively associated with household income.

**Table 2:** Association of individual and neighbourhood characteristics with leisure-time walking and cycling

	**% N**	**Odds Ratio (95% CI) Model 3**^**a**^	
		**Leisure-time walking**	**Leisure-time cycling**
**Individual characteristics**			
**Age (years)**	49 ± 17	1.01 (1.01 – 1.01)*	1.00 (1.00 – 1.00)
**Gender**			
Men	47.2%	1.00	1.00
Women	52.8%	1.46 (1.38 – 1.55)*	1.18 (1.12 – 1.25)*
**Ethnicity**			
Native (Dutch)	87.1%	1.00	1.00
Non-native, Western	6.4%	1.19 (1.06 – 1.35)*	0.84 (0.75 – 0.95)*
Non-native, non-Western	3.5%	1.16 (0.99 – 1.37)	0.54 (0.45 – 0.63)*
Non-native, origin unknown	2.9%	0.90 (0.76 – 1.06)	0.68 (0.58 – 0.81)*
**Household composition**			
Married/partner, no children	37.8%	1.00	1.00
Married/partner with child(ren)	39.9%	0.93 (0.87 – 1.01)	0.94 (0.87 – 1.01)
Single, no children	17.2%	0.74 (0.68 – 0.81)*	0.77 (0.71 – 0.84)*
Single with child(ren)	4.4%	0.70 (0.60 – 0.81)*	0.75 (0.65 – 0.87)*
Unknown	0.8%	0.59 (0.43 – 0.80)*	0.77 (0.56 – 1.06)
**Education**			
Tertiary education	26.3%	1.00	1.00
Secondary education: upper level	35.5%	0.73 (0.67 – 0.79)*	0.79 (0.74 – 0.86)*
Secondary education: mid level	8.5%	0.65 (0.58 – 0.73)*	0.73 (0.65 – 0.82)*
Secondary education: lower level	14.8%	0.58 (0.52 – 0.64)*	0.73 (0.66 – 0.81)*
Primary education	14.6%	0.42 (0.38 – 0.47)*	0.48 (0.43 – 0.53)*
Unknown	0.3%	1.08 (0.61 – 1.91)	0.63 (0.38 – 1.06)
**Household income (€)**			
>29.900	20.9%	1.00	1.00
23.600 – 29.900	20.5%	1.02 (0.93 – 1.12)	1.04 (0.95 – 1.14)
19.200 – 23.600	20.1%	1.00 (0.91 – 1.10)	1.15 (1.04 – 1.26)*
15.200 – 19.200	19.8%	1.03 (0.93 – 1.13)	1.21 (1.09 – 1.33)*
<15.200	17.8%	0.95 (0.85 – 1.05)	1.11 (0.99 – 1.23)
Unknown	1.1%	0.93 (0.70 – 1.25)	1.06 (0.79 – 1.41)
**Household wealth (€)**			
>293.469	15.1%	1.00	1.00
148.000 – 293.469	15.0%	0.97 (0.87 – 1.09)	0.97 (0.87 – 1.08)
39.047 – 148.000	14.5%	1.02 (0.92 – 1.14)	0.87 (0.78 – 0.97)*
3.362 – 39.047	13.6%	0.89 (0.80 – 1.00)	0.71 (0.63 – 0.79)*
<3.362	12.2%	1.00 (0.89 – 1.13)	0.70 (0.62 – 0.79)*
Unknown	29.6%	1.02 (0.92 – 1.13)	0.83 (0.75 – 0.91)*
**Neighbourhood characteristics**			
**Population density**			
Very dense	16.5%	1.00	1.00
Dense	27.6%	0.92 (0.84 – 1.01)	0.99 (0.90 – 1.09)
Moderately dense	21.8%	0.97 (0.88 – 1.08)	1.03 (0.93 – 1.14)
Slightly dense	23.2%	0.99 (0.89 – 1.10)	1.04 (0.94 – 1.16)
Not dense	10.9%	1.03 (0.91 – 1.17)	0.97 (0.85 – 1.10)

*p≤0,05.

^a^Adjusted for age, gender, ethnicity, household composition, education, household income, household wealth, population density.

Table 3 shows that neighbourhood variations in levels of safety were most pronounced for traffic safety. The four specific safety components were highly to moderately correlated with each other, except for traffic safety.

**Table 3:** Mean neighbourhood-level safety scores and the correlation between safety variables

**Safety variables**	**Mean (SD)**	**Range**	**Pearson correlation coefficient**			
			**General safety**	**Physical disorder**	**Social disorder**	**Crime-related fear**
General safety	4.35 (0.28)	2.81 – 4.97				
Physical disorder	4.24 (0.35)	2.83 – 4.95	0.80			
Social disorder	4.49 (0.30)	3.15 – 4.96	0.76	0.68		
Crime-related fear	4.65 (0.37)	2.08 – 4.96	0.73	0.56	0.53	
Traffic-safety	4.01 (0.50)	1.67 – 4.89	0.66	0.28	0.24	0.17

**Figure 1:**
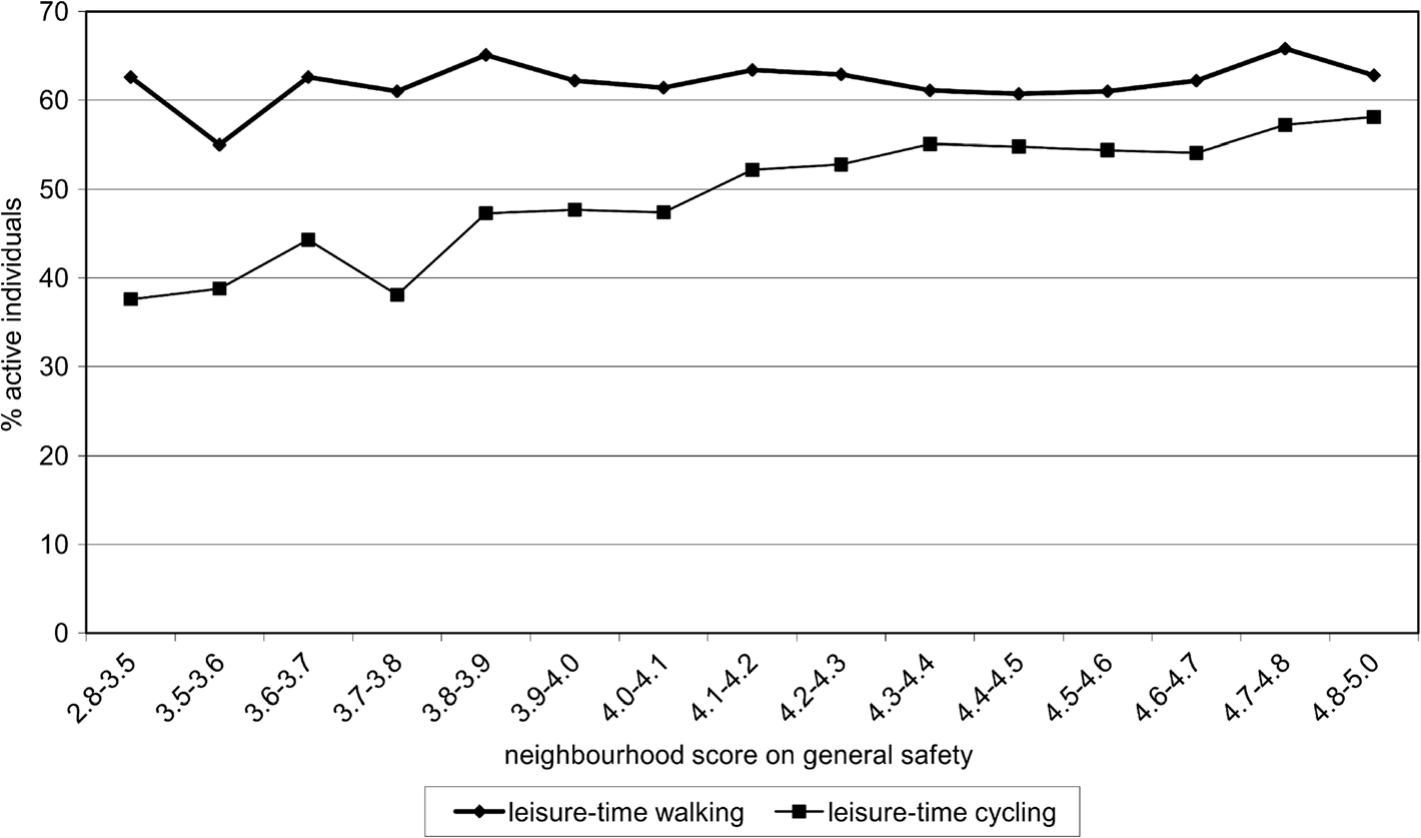
Weighted percentage of individuals engaging in leisure-time walking or cycling for at least 30 minutes per week, by level of general neighbourhood safety.

Figure 1 shows to what amount the prevalence of leisure-time PA varied according to the level of general neighbourhood safety. The prevalence of leisure-time walking remained the same (60%) across the range of general safety, while prevalence of leisure-time cycling increased from 40% in the most unsafe to 60% in the safest neighbourhoods.

Logistic regression analyses also show no strong association between neighbourhood safety and leisure-time walking (Table 4). The association was not statistically significant for general safety and the four specific safety components. In contrast, for leisure-time cycling, a positive and statistically significant association was found with both general neighbourhood safety and each of the four specific safety components. A 1-unit increase in general neighbourhood safety was associated with a 40% increase in odds of leisure-time cycling. Associations with the specific safety components were somewhat weaker, and weakest for traffic safety. With a 1-unit increase in neighbourhood traffic safety, odds of leisure-time cycling increased by only 9%. For all safety indicators, odds ratios of leisure-time walking and cycling changed to only a small extent after potential confounders were added to the model. Thus, measurable confounders appeared to have only a limited impact on the observed associations. The ICC shows that nearly one percent of the variance in leisure-time cycling is attributable to the neighbourhood-level, while there is no statistical support for between-neighbourhood differences in walking (Table 4).

**Table 4:** Association of general safety and specific safety components with leisure-time walking and -cycling

	**Odds ratio (95% CI)**		
	**Model 1 (age, gender, ethnicity, household comp.)**	**Model 2 (+education, income, wealth)**	**Model 3 (+population density)**
**Leisure-time walking**			
General safety	0.96 (0.86 – 1.08)	0.95 (0.85 – 1.06)	0.92 (0.81 – 1.05)
Physical disorder	0.94 (0.86 – 1.03)	0.94 (0.86 – 1.03)	0.90 (0.81 – 1.01)
Social disorder	0.94 (0.84 – 1.04)	0.95 (0.85 – 1.05)	0.92 (0.82 – 1.04)
Crime-related fear	0.98 (0.90 – 1.06)	0.98 (0.90 – 1.06)	0.97 (0.88 – 1.06)
Traffic safety	1.02 (0.95 – 1.09)	1.00 (0.94 – 1.07)	1.00 (0.93 – 1.06)
**Leisure-time cycling**			
General safety	1.50 (1.34 – 1.68)*	1.42 (1.27 – 1.59)*	1.40 (1.23 – 1.60)*
Physical disorder	1.34 (1.22 – 1.47)*	1.28 (1.17 – 1.41)*	1.27 (1.14 – 1.42)*
Social disorder	1.31 (1.18 – 1.46)*	1.27 (1.14 – 1.41)*	1.22 (1.08 – 1.37)*
Crime–related fear	1.29 (1.19 – 1.40)*	1.26 (1.16 – 1.37)*	1.23 (1.12 – 1.35)*
Traffic safety	1.13 (1.05 – 1.20)*	1.10 (1.04 – 1.18)*	1.09 (1.02 – 1.17)*
**Intraclass Correlation Coefficient**			
Leisure-time walking	0.00 (0.00 – 0.04)	0.00 (0.00 – 0.62)	0.00 (0.00 – 0.99)
Leisure-time cycling	0.01 (0.01 – 0.02)*	0.01 (0.00 – 0.02)*	0.01 (0.00 – 0.02)*

* p≤0.05.

Subpopulation differences in associations between general safety and leisure-time walking and cycling are presented in Table 5. For leisure-time walking, a significant inverse association with general safety was found for the youngest and oldest men and for the lower educated individuals. In these groups, an increase in general neighbourhood safety was related to a decrease in odds of leisure-time walking. For leisure-time cycling, the association with general neighbourhood safety was found to strengthen with increasing age. The association was also stronger for women compared to men of the same age. Cycling behaviour is most strongly related to safety for older women. The association between general safety and cycling appeared to be strongest with very dense populations, although interaction with population density was not statistically significant.

**Table 5:** Association of general safety with leisure-time walking and cycling in different subgroups

	**Leisure-time walking**		**Leisure-time cycling**	
	**Odds ratio (95% CI)**	**p-value of interaction**	**Odds ratio (95% CI)**	**p-value of interaction**
**Age-gender category**				
Men 18–30 years	0.42 (0.27 – 0.65)*	Reference	1.25 (0.80 – 1.94)	Reference
Men 31–45 years	0.90 (0.67 – 1.21)	<0.01*	1.25 (0.93 – 1.69)	0.99
Men 46–65 years	1.15 (0.87 – 1.51)	<0.01*	1.37 (1.04 – 1.80)*	0.72
Men > 65 years	0.65 (0.45 – 0.93)*	0.14	1.53 (1.06 – 2.19)*	0.48
Women 18–30 years	1.12 (0.76 – 1.64)	<0.01*	1.01 (0.69 – 1.48)	0.47
Women 31–45 years	1.20 (0.90 – 1.60)	<0.01*	1.39 (1.05 – 1.84)*	0.68
Women 46–65 years	1.06 (0.80 – 1.42)	<0.01*	1.74 (1.32 – 2.30)*	0.20
Women > 65 years	1.02 (0.73 – 1.43)	<0.01*	2.23 (1.57 – 3.16)*	0.04*
**Educational level**^**a**^				
Higher educated	1.04 (0.88 – 1.22)	Reference	1.37 (1.17 – 1.61)*	Reference
Lower educated	0.79 (0.66 – 0.95)*	0.02*	1.48 (1.22 – 1.78)*	0.51
**Population density**				
Very dense	0.98 (0.75 – 1.27)	Reference	1.70 (1.30 – 2.23)*	Reference
Dense	0.96 (0.76 – 1.21)	0.91	1.33 (1.05 – 1.68)*	0.17
Moderately dense	0.89 (0.67 – 1.19)	0.64	1.41 (1.06 – 1.88)*	0.34
Slightly dense	0.84 (0.60 – 1.18)	0.50	1.24 (0.88 – 1.74)	0.15
Not dense	0.86 (0.55 – 1.34)	0.62	1.23 (0.79 – 1.92)	0.22

* p≤0,05.

^a^Higher educated: tertiary education, upper level secondary education.

Lower educated: mid and lower level secondary education, primary education.

## Discussion

In the present study, an increase in general neighbourhood safety and all specific safety components was found to be associated with an increase in leisure-time cycling participation. Associations were strongest for general safety and among older women. Overall, none of the neighbourhood safety outcomes was significantly associated with leisure-time walking. However, an increase in general neighbourhood safety was found to be associated with a decrease in leisure-time walking participation for the youngest and oldest adult men and for the lower educated people.

### Limitations

The data available to this study had some potential limitations. First, while many potential confounders at the individual level have been accounted for, not all neighbourhood-level factors have taken into account that may have confounded the association between safety and PA. For example, an area with mixed land use and nearby shopping and recreational facilities may stimulate leisure-time walking, while such an area might at the same time be relatively unsafe. In the present study, confounding by such factors may have concealed an association between safety and leisure-time walking. In order to address this potential source of bias, we have adjusted our analyses for population density, as this variable may correlate with land use mix and the proximity of facilities. We found that control for population density had negligible effects on the associations between safety and walking or cycling. Future research should aim to control for neighbourhood-level confounders in more detail.

There was a non-response of 36% for the POLS interview and another 16% for the additional paper-and-pencil survey. Non-respondents may have differed in important aspects from the study population. However, this would only have biased the results of this study if non-response varied according to the level of individual PA, as well as the degree of neighbourhood safety, after accounting for confounders. We think this is not very likely, but we can not entirely exclude the possibility of some bias.

Another limitation has to do with the self-reported nature of PA. People may have difficulty estimating their time spent on PA and may be inclined to give socially desirable answers, which might have caused them to overestimate their levels of PA [31]. However, this only becomes a problem if the amount of overestimation differs between safe and unsafe neighbourhoods after controlling for individual-level confounders. Again, we think this is not very likely.

Neighbourhood safety was also self-reported. We chose to use subjective measures of neighbourhood safety, because there is evidence that objective and subjective ratings are poorly correlated [16,32] and that subjective ratings may be more important in determining PA behaviour [32,33]. Those in favour of objective measures, have argued that objective measures are needed to avoid bias associated with self-reports, such as the ‘single source bias’ [34]. This source of bias may occur if self-reported data on both determinants and outcomes are collected using the same survey. To avoid this source of bias, we have used two different sets of respondents for measuring neighbourhood safety (the WoON survey) and leisure-time walking and cycling (the POLS survey).

### Interpretation of key results

Associations between safety and leisure-time cycling have been found to be strongest for general safety. This suggests that a combination of safety problems may have more impact on people’s engagement in cycling than isolated safety problems. The weakest association with cycling has been found for traffic safety. This supports an earlier study that found traffic safety to have less influence on leisure-time PA than other safety components [6]. The weak association for traffic safety is remarkable as neighbourhood variations in levels of safety were most profound for traffic safety (Table 2). Results of the present study suggest that people are more influenced by social aspects of safety such as crime and social disorder, than by traffic safety.

Subgroup analyses suggested that elderly women were most influenced by general neighbourhood safety in their cycling behaviour. This supports earlier research [22,35]. Foster et al. [22] suggest that women and elderly may be more sensitive to safety as they feel more physically vulnerable than men and young adults.

The positive association between neighbourhood safety and cycling may in part reflect a safety component that has not been included: fear of bicycle theft or damage. This seems to be a plausible mechanism since bicycle theft rates among bicycle owners in the Netherlands are the highest in the world [24].

Leisure-time walking was not significantly associated with general neighbourhood safety or any of its safety components. An American study that used various objectively measures safety components, yielded the same results [6]. Many other studies, too, did not find an association between safety and leisure-time walking [6-13,17,20,21]. Some evidence of an association was found in other studies, but results were mostly inconsistent [4,5,14-16,18,19].

Lack of a positive association in the present study might have been due to focus on fairly large neighbourhoods. 4-digit zip code areas have an average of 4.088 inhabitants [36]. Environmental influences of safety on walking may have been more apparent on a smaller scale, since walking is primarily performed in the immediate area around the home. Future research should assess if a focus on smaller areas reveals a more significant association.

Subgroup analyses have shown an inverse relationship between safety and leisure-time walking for young men and for lower educated. Earlier studies support this finding [18,37,38]. Ross et al. [38] suggest that unsafe neighbourhoods have a culture of being outside on the streets, which may be especially attractive to these groups.

## Conclusions

The current study indicates that, in the Netherlands, a safe neighbourhood may increase adults’ participation in leisure-time cycling but not necessarily in walking. Leisure-time cycling may best be encouraged by improving different safety components at once, rather than focusing on one aspect such as traffic safety. Special attention needs to be paid to residents that are particularly sensitive to the safety situation in the neighbourhood, such as older women.

## Competing interests

The authors declare that they have no competing interests.

## Authors’ contributions

DK and AK developed the study design. MW and DK prepared the data. DK performed the analyses and, with help of AK, drafted the manuscript. JM and MW revised intermediate results and manuscript versions and made substantial contributions to subsequent versions. All authors have read and approved the final version of the manuscript.
